# Prognostic role of pretreatment ^18^F-FDG PET/CT and hematological parameters in relapsed/refractory Hodgkin lymphoma patients treated with immune checkpoint inhibitors and chemotherapy: a dual-center cohort study

**DOI:** 10.1186/s12880-023-00967-x

**Published:** 2023-01-21

**Authors:** Tianyu Yang, Shuang Liu, Rui Zuo, Hongwei Liang, Lu Xu, Zhengjie Wang, Xiaoliang Chen, Hua Pang

**Affiliations:** 1 Department of Nuclear Medicine, The First Affiliated Hospital of Chongqing Medical University, Chongqing, 400042 China; 2 Department of Radiology, The First Affiliated Hospital of Chongqing Medical University, Chongqing, 400042 China; 3 Department of Nuclear Medicine, Chongqing University Cancer Hospital, Chongqing, 400030 China

**Keywords:** Immune checkpoint inhibitors, Chemotherapy, Classical Hodgkin lymphoma, ^18^F-FDG PET/CT, Prognosis

## Abstract

**Background:**

The combination of anti-programmed death-1 antibodies and chemotherapy is effective; however, there are no reliable outcome prediction factors. We investigated the prognostic factors based on ^18^Fluorine-fluorodeoxyglucose positron emission tomography/computed tomography (^18^F-FDG PET/CT) quantitative and hematological parameters to predict progression-free survival (PFS) in relapsed/refractory classical Hodgkin lymphoma (R/R cHL) patients treated with immune checkpoint inhibitors (ICIs) and chemotherapy.

**Methods:**

This retrospective study included 31 patients who underwent ^18^F-FDG PET/CT before and during treatment. Pretreatment metabolic and hematological parameters were evaluated using Cox regression analysis to identify predictors of PFS. Based on the cut-off values calculated using the receiver operating characteristic (ROC) curve, patients were classified into low-, intermediate-, and high-risk groups. Kaplan–Meier curves and the log-rank test were used to compare survival differences between the groups.

**Results:**

Cox multivariable analysis indicted that the treatment response based on Lactate dehydrogenase (LDH), Lugano classification and SUV_max_ were independent predictors of PFS (*P* = 0.004, 0.007 and 0.039, respectively). The optimal cut-off values for SUV_max_ and LDH were 11.62 and 258.5 U/L, respectively (*P* < 0.01). Survival curves showed that LDH ≥ 258.5U/L and SUV_max_ ≥ 11.62 were correlated to shorter PFS (*P* < 0.001, *P* = 0.003, respectively). The differences in PFS between the low-, intermediate-, and high-risk groups were statistically significant (*P* = 0.0043).

**Conclusion:**

In R/R cHL patients treated with ICIs and chemotherapy, Lugano classification, SUV_max_, and LDH were significantly correlated with PFS. The combination of metabolic and hematological parameters predicts PFS and may help to improve patient selection.

## Introduction

Anti-programmed death-1 (anti-PD-1) antibodies have a high overall response rate (ORR: 65–87%) in patients with relapsed/refractory classical Hodgkin lymphoma (R/R cHL) after autologous stem cell transplantation and brentuximab vedotin treatment. However, a complete response (CR) occurs in only 9–22% of patients [1, 2]. The median progression-free survival (PFS) is 11–15 months, and a long-term response is observed in only a few patients [3–5]. The combination of anti-PD-1 antibodies and chemotherapy is associated with an ORR of 87–100% and a median PFS of 35 months [6–8]. This indicates that treatment with immune checkpoint inhibitors (ICIs) and chemotherapy may be suitable for R/R cHL patients.

It is important to accurately predict the patients’ prognosis because immunotherapy may lead to unique treatment responses, including pseudoprogression and hyperprogression. Although various risk factors have been investigated to predict R/R cHL prognosis in patients treated with ICIs and chemotherapy [6, 7], few of these were based on imaging. ^18^Fluorine-fluorodeoxyglucose positron emission tomography/computed tomography (^18^F-FDG PET/CT) has various advantages in cHL diagnosis, staging, treatment response and prognosis evaluations. Pretreatment ^18^F-FDG PET/CT metabolic parameters, including the standardized uptake value (SUV), metabolic tumor volume (MTV), and total lesion glycolysis (TLG), have prognostic significance in cHL patients [9–11]. MTV is a measurable volumetric parameter that is calculated by a set threshold value for lesions with high FDG uptake; TLG is based on MTV and is a comprehensive parameter that represents the metabolic activity and metabolic volume of the tumor. Previous studies have suggested that unfavorable outcomes of immunotherapy are related to the pro-inflammatory status of patients [12, 13]. Hematological parameters such as lactate dehydrogenase (LDH) and the derived neutrophil-to-lymphocyte ratio (dNLR) may predict poor outcomes in cHL patients [13].

To date, studies about the prognostic role of ^18^F-FDG PET/CT metabolic parameters in R/R cHL patients treated with anti-PD-1 combination therapy are lacking. We hypothesized that the combination of unfavorable pretreatment metabolic and hematological parameters may be related to a poor cHL prognosis.

## Materials and methods

This study was approved by the Ethics Committees of the First Affiliated Hospital of Chongqing Medical University (2022-K180) and Chongqing University Cancer Hospital (CZLS2022127-A). Written informed consent was obtained from all the patients prior to the treatment.

### Patients

The study retrospectively included patients treated with ICIs and chemotherapy who underwent pretreatment ^18^F-FDG PET/CT at Chongqing Medical University and Chongqing University Cancer Hospital between January 2018 and December 2021. The inclusion criteria were as follows: (1) at least one FDG-avid lesion, defined as a lesion with a higher FDG uptake than the liver; and (2) at least one treatment response evaluation. The exclusion criteria were as follows: (1) ICIs monotherapy; (2) no available pretreatment and follow-up imaging data; (3) < 18 years; (4) no FDG-positive lymphoma lesion; and (5) history of other malignant tumors.

Eighteen patients were treated with anti-PD-1 monoclonal antibodies (mAbs) + ABVD (Anti-PD-1 mAbs: 200 mg, day 1; Doxorubicin: 25 mg/m^2^, day 2, day 16; Bleomycin: 10 mg/m^2^, day 2, day 16; Vinblastine: 1.4 mg/m^2^, day 2, day 16; Dacarbazine: 375 mg/m^2^, day 2, day 16); 7 with anti-PD-1 mAbs + DHAP (Anti-PD-1 mAbs: 200 mg, day 1; Cisplatin: 100 mg/m^2^, day 2; Cytarabine: 2 g/m^2^, day 3; Dexamethasone: 40 mg, day 2-day 5); 5 with anti-PD-1 mAbs + BEACOPP (Anti-PD-1 mAbs: 200 mg, day 1; Bleomycin: 10 mg/m^2^, day 9; Etoposide: 100 mg/m^2^, day 2-day 4; Doxorubicin: 25 mg/m^2^, day 2; Cyclophosphamide: 650 mg/m^2^, day 2; Vincristine: 1.4 mg/m^2^, day 9; Procarbazine: 100 mg/m^2^, day 2-day 8; Prednisone: 40 mg/m^2^, day 2-day 15) and 1 with anti-PD-1 mAbs + BV (Brentuximab Vedotin: 1.8 mg/kg day 1; Anti-PD-1 mAbs: 200 mg, day 8).

### Data collection

Hematological parameters within 3 days before the start of ICIs combination treatment were acquired from the medical records. The parameters included: age, sex, staging, international prognostic score (IPS), histology, B symptoms, Epstein‒Barr virus (EBV), hepatitis B virus (HBV), bulky disease (defined as a maximum extra or intranodal lesion width of ≥ 10 cm on imaging), LDH levels (reference range: 120–250 U/L), absolute neutrophil and platelet counts, albumin level, and dNLR (dNLR = absolute neutrophil count/[white blood cell concentration − absolute neutrophil count]).

### PET/CT image acquisition

Pretreatment PET/CT scans were performed within 14 days before immunochemotherapy. The timing of the PET/CT scans for treatment response evaluation was scheduled by the hematologic oncologists. Images were acquired in accordance with the European Association of Nuclear Medicine guidelines [14]. ^18^F-FDG PET/CT was performed after at least 6 h of fasting and patients had to have a blood glucose level of ≤ 6.1 mmol/L. Skull base to mid-thigh images were acquired 60 min after the intravenous administration of 3.70–5.55 MBq/kg of ^18^F-FDG using a Philips Gemini TF 64 PET/CT tomograph (Philips Medical Systems, Andover, MA, USA) or a Discovery 710 PET/CT tomograph (General Electric Co., Boston, MA, USA) with standard parameters (CT: 100 mAs, 120 keV, 4.0 mm/slice thickness, and 512 × 512 matrix; PET: 4.0 mm/slice thickness, 2.5–4 min/bed, and 128 × 128 matrix). Order-subset expectation maximization was performed after attenuation correction (2 iterations and 8 subsets) for image reconstruction.

### Image analysis

Anonymized PET/CT images were transferred to a core nuclear medicine center for analysis. Two experienced nuclear medicine physicians with at least 5 years of experience in ^18^F-FDG PET/CT evaluation and lymphoma imaging visually and semiquantitatively analyzed the PET/CT images. Suspected lesions were discussed by the two physicians to reach a consensus. The regions of interest were manually drawn on hypermetabolic lesions (i.e., lesions with greater FDG uptake than the liver) using the MEMRS Workstation (MedEx China Co., Ltd., Beijing, China). The maximum, mean, and peak SUV (SUV_max_, SUV_mean_, and SUV_peak_, respectively), MTV (isocontour threshold method based on 41% SUV_max_), and TLG values were obtained. The whole-body tumor burden values of SUV_max_, SUV_mean_, SUV_peak_, MTV, and TLG (SUV_maxwb_, SUV_meanwb_, SUV_peakwb_, MTV_wb_, and TLG_wb_, respectively) were calculated as the sums of the nodal and extranodal lesions.

### Treatment response evaluation

PET/CT and CT were evaluated by two nuclear physicians experienced in lymphoma imaging. Based on the Lugano classification and Lymphoma Response to Immunomodulatory therapy Criteria (LYRIC, 2016), the patients were categorized into CR, partial response (PR), stable disease (SD), and progressive disease (PD) groups. A CR was classified as clinical benefit (CB), while PR, SD, and PD were classified as “no clinical benefit” (NCB). LYRIC also includes the category of indeterminate response (IR) for first-time PD, so we classified PD on PET/CT as IR1–3.

### Study endpoint

The primary endpoint was PFS, defined as the time between treatment initiation and the date of relapse or progression or the date of last follow-up. Survival time was calculated from the date of ICIs combination therapy initiation to the date of relapse or progression or final follow-up. Based on LYRIC, the last unconfirmed PD before subsequent confirmation or the end of follow-up was defined as disease progression.

### Statistical analysis

SPSS Statistics (version 25.0; IBM Corp., Armonk, NY, USA), GraphPad Prism (version 9.1.0; GraphPad Software Inc., San Diego, CA, USA), and R (version 4.1.2; R Foundation for Statistical Computing, Vienna, Austria) software were used for statistical analysis. Descriptive statistics are reported as medians (interquartile range [IQR]). The Mann‒Whitney U test was used to compare baseline metabolic and hematological parameters between the CB and NCB groups. Cox regression analysis was used to identify the variables independently correlated with survival. Variables with *P* < 0.05 in the univariable analyses were included in the multivariable survival analysis. Receiver operating characteristic (ROC) curves were used to identify the optimal cutoff values for the parameters. Kaplan‒Meier curves and the log-rank test were used to compare survival differences between the groups. Median follow-up time was evaluated by the reverse Kaplan‒Meier method. *P* < 0.05 were considered statistically significant.

## Results

### Patients’ characteristics

**Fig. 1:**
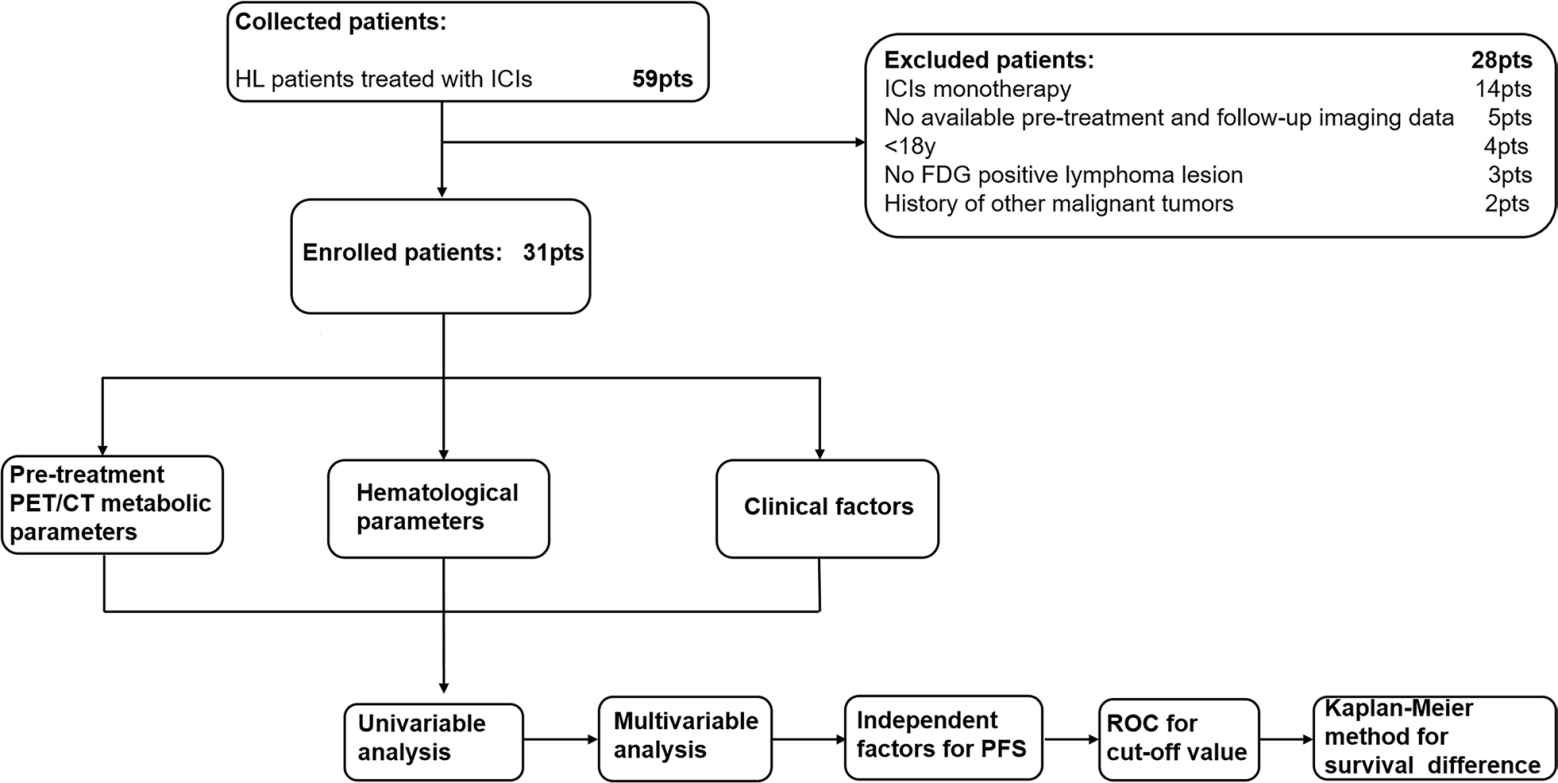
Study flowchart

Between January 2018 and December 2021, 59 cHL patients treated with ICIs at two nuclear medicine centers were retrospectively enrolled, of whom 31 were included in this study. The remaining 28 patients did not meet the inclusion criteria (Fig. 1). Demographic and baseline data of the patients are presented in Table 1. The patients were predominantly males (80.6%, 25/31) with a median age of 32 years (range: 19–74 years). The Ann Arbor system was used for tumor staging (stage I: *n* = 0; stage II: *n* = 6, 19.3%; stage III: *n* = 11, 35.5%; and stage IV: *n* = 14, 45.2%). IPS were < 3 and ≥ 3 for 16 (51.6%) and 15 (48.4%) patients, respectively. Among these patients, 22 had nodular sclerosis, 12 had mixed cellularity, and 3 had lymphocyte enrichment; there were no cases of lymphocyte depletion. Nodular sclerosis was the most common feature and was present in 6 (60%) and 10 (47.6%) patients in the CB and NCB groups, respectively. B symptoms were present in 19 (61.3%) patients, of whom 4 (40%) and 15 (71.4%) belonged to the CB and NCB groups, respectively. EBV and HBV were detected in 13 (41.9%) and 6 (19.4%) patients, respectively. Bulky disease was present in 3 patients in each group (CB: 30%, NCB: 14.2%).

**Table 1:** Baseline characteristics of 31 patients in CB (Clinical Benefit) and NCB (no- Clinical Benefit) group

Characteristic	Total (n = 31)	CB (n = 10)	NCB (n = 21)
Age			
Median age, year(range)	32 (19–74)	31 (20–66)	32 (19–74)
Sex n. (%)			
Male	25 (80.6)	9 (90.0)	16 (76.2)
Female	6 (19.4)	1 (10.0)	5 (23.8)
Staging n. (%)			
I	NA	NA	NA
II	6 (19.3)	1 (10.0)	5 (23.8)
III	11 (35.5)	3 (30.0)	8 (38.1)
IV	14 (45.2)	6 (60.0)	8 (38.1)
IPS			
< 3	16 (51.6)	7 (70.0)	14 (66.7)
≥ 3	15 (48.4)	3 (30.0)	7 (33.3)
Histology n. (%)			
Nodular sclerosis	16 (51.6)	6 (60.0)	10 (47.6)
Mixed cellularity	12 (38.7)	3 (30.0)	9 (42.9)
Lymphocyte rich	3 (9.7)	1 (10.0)	2 (9.5)
Lymphocyte depletion	NA	NA	NA
B symptoms	19 (61.3)	4 (40.0)	15 (71.4)
EBV	13 (41.9)	4 (40.0)	9 (42.9)
HBV	6 (19.4)	2 (20.0)	4 (19.0)
Bulky disease	6 (19.4)	3 (30.0)	3 (14.2)

Staging was following Ann Arbor-Cotswolds system; *IPS* international prognostic score; *EBV* Epstein-Barr virus; *HBV* hepatitis B virus; *NA* not available

### Treatment response

PET/CT scans for treatment response evaluation was performed at a median of 2.3 months (IQR, 1.5–3.8 moths) after treatment initiation. Based on the Lugano classification, there were 10 (32.3%; CR) and 21 (67.7%; SD/PR: 13/31, 41.9% and PD: 8/31, 25.8%) patients in the CB and NCB groups, respectively.

Metabolic parameters were obtained on pretreatment PET/CT performed within 14 days of starting immunochemotherapy. Hematological were collected from medical records within 3 days before the start of ICIs combination treatment. These parameters were compared between the groups using the Mann‒Whitney U test (Table 2).

**Table 2:** Mann–Whitney U test for differences in groups

Pretreatment parameters	CB (n = 10) [median (IQR)]	NCB (n = 21) [median (IQR)]	*P* value
SUV_max_	5.36 (4.26, 10.20)	12.50 (8.26, 15.78)	0.004*
SUV_mean_	2.03 (1.55, 4.20)	3.87 (2.50, 5.35)	0.043*
SUV_peak_	3.45 (2.41, 9.73)	8.59 (6.38, 12.74)	0.017*
MTV	3.65 (1.78, 9.52)	11.65 (5.51, 32.00)	0.065
TLG	8.89 (3.42, 49.74)	47.01 (23.90, 422.73)	0.053
SUV_maxwb_	51.57 (4.73, 82.69)	112.50 (30.26, 302.32)	0.031*
SUV_meanwb_	24.48 (2.25, 49.72)	65.68 (14.80, 157.55)	0.043*
SUV_peakwb_	37.42 (3.09, 58.21)	85.76 (23.18, 216.98)	0.053
MTV_wb_	29.21 (2.37, 145.26)	111.14 (21.79, 531.84)	0.035*
TLG_wb_	61.15 (4.99, 303.46)	263.86 (64.24, 1644.70)	0.035*
LDH, U/L	226.00 (186.00, 240.50)	322.00 (275.00, 359.50)	0.001*
Absolute platelet count, × 10^9^/L	248.00 (187.25, 275.00)	188.00 (141.00, 308.50)	0.393
Absolute neutrophil count, × 10^9^/L	4.22 (3.04, 5.27)	3.87 (2.14, 4.84)	0.693
Albumin levels, g/L	45.67 (42.25, 49.02)	44.00 (38.15, 51.90)	0.852
dNLR	2.59 (1.87, 3.71)	1.78 (1.09, 3.27)	0.186

*CB* Clinical Benefit; *NCB* no-Clinical Benefit; *IQR* interquartile range; *SUV*_*max*_ maximum standardized uptake value; *SUV*_*mean*_ mean standardized uptake value; *SUV*_*peak*_ peak standardized uptake value; *MTV* metabolic tumor volume; *TLG* total lesion glycolysis; *SUV*_*maxwb*_ whole-body maximum standardized uptake value; *SUV*_*meanwb*_ whole-body mean standardized uptake value; *SUV*_*peakwb*_ whole-body peak standardized uptake value; *MTV*_*wb*_ whole-body metabolic tumor volume; *TLG*_wb_ whole-body total lesion glycolysis; *LDH* lactate dehydrogenase; *dNLR* derived neutrophil-to-lymphocyte ratio, defined as absolute neutrophil count/[white blood cell concentration − absolute neutrophil count];*P < 0.05

The NCB group had significantly higher LDH, SUV_max_, SUV_peak_, SUV_maxwb_, MTV_wb_, TLG_wb_, SUV_mean_ and SUV_meanwb_ values than the CB group (*P* = 0.001, 0.004, 0.017, 0.031, 0.035, 0.035, 0.043 and 0.043, respectively), indicating a high tumor burden in the NCB group.

### Survival analysis

**Fig. 2:**
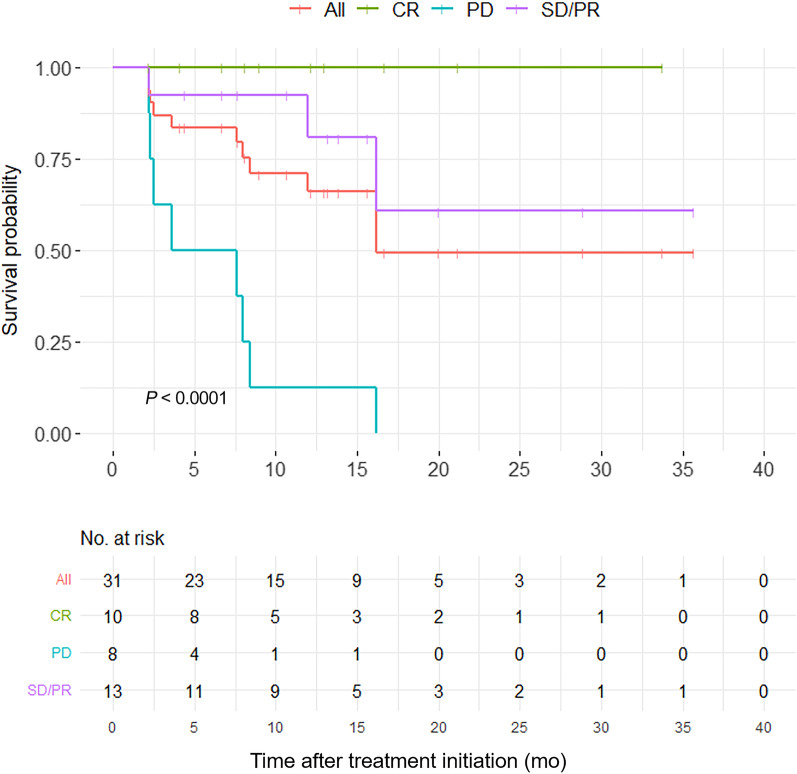
Kaplan–Meier estimate of PFS according to Lugano classification. In the whole cohort (31 patients), patients were stratified into 3 groups: CR, SD/PR and PD. SD and PR were grouped together as there was only one SD patient in the study and the PFS was similar to PR patients

The reverse Kaplan‒Meier method showed that disease progression occurred in 11 patients at a median follow-up time of 13.2 months (IQR = 8.1–21.2). The median and mean PFS durations were 16.2 months (95% confidence interval [CI] = 3.76–28.63) and 21.2 months (95% CI = 15.47–26.97), respectively. The estimated 18-, 12-, and 6-month PFS rates were 49.4% (95% CI = 0.30–0.81), 65.9% (95% CI = 0.50–0.88) and 83.5% (95% CI = 0.71–0.99), respectively (Fig. 2).

For patients with CR, PR and SD, 10/10 CR patients had no FDG-avid lesions at the last follow-up. There were 3/13 PR and SD patients who experienced disease progression during follow-up. According to LYRIC, IR2-3 patients (*n* = 8) were confirmed to have PD during follow-up.

A survival curve was used to assess the PFS for the four Lugano classifications (*P* < 0.01) (Fig. 2). The median PFS for PD was 5.6 months, while that for CR and SD/PR could not be determined. The SD and PR groups were combined because there was only one patient had SD with similar PFS to PR patients.

**Fig. 3:**
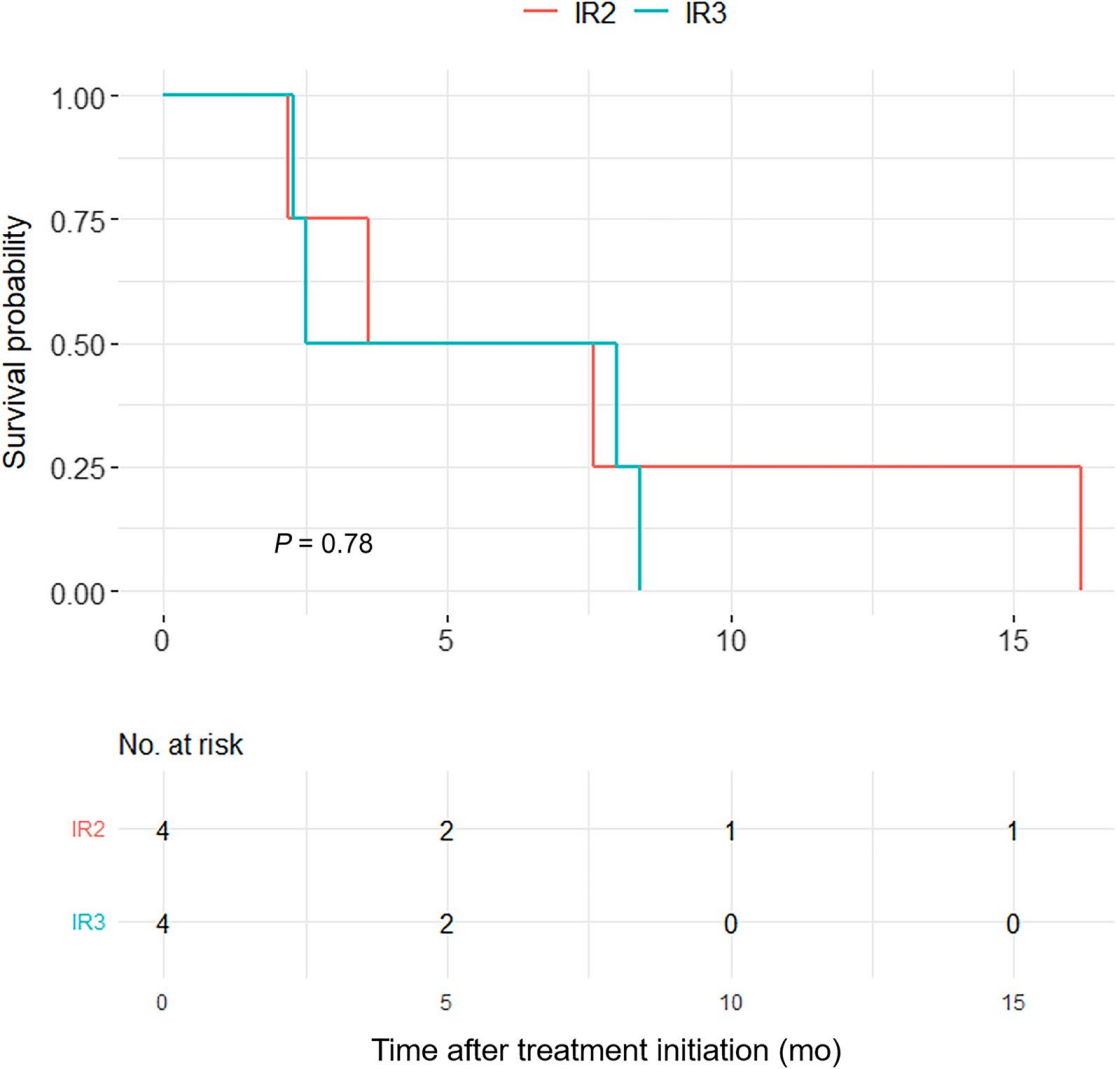
Kaplan–Meier estimate of PFS in IR2 and IR3 patients according to LYRIC

Based on LYRIC, the 8 PD patients were further categorized as IR1–3. There were no IR1 patients (≥ 50% increase in the sum of the products of diameters in the first 12 weeks), and there were 4 (50%) IR2 (≥ 50% lesion growth without overall progression [< 50% increase]) and 4 (50%) IR3 (increased ^18^F-FDG uptake without increased lesion size or number) patients each. The median PFS durations were 2.5 and 3.6 months for IR3 and IR2 patients, respectively (*P* = 0.78) (Fig. 3).

Cox univariable and multivariable regression analyses of PFS in R/R cHL patients indicated that the Lugano classification, SUV_max_, and LDH were significant prognostic factors (Table 3).

**Table 3:** Univariable analysis and multivariable analysis for PFS

Variable	Univariable analysis		Multivariable analysis	
	HR (95%CI)	*P* value	HR (95%CI)	*P* value
Sex	0.034 (0.000–17.840)	0.291		
Age	0.995 (0.949–1.043)	0.829		
Histology	1.058 (0.442–2.531)	0.899		
Lugano classification	9.421 (2.070–32.784)	< 0.001*	12.245(1.999–75.008)	0.007*
Staging	0.627 (0.538–2.797)	0.627		
IPS	1.097 (0.714–1.686)	0.671		
B symptoms	2.390 (0.514–11.112)	0.267		
EBV	1.469 (0.441–4.892)	0.531		
HBV	1.020 (0.219–4.743)	0.980		
Bulky disease	0.472 (0.060–3.710)	0.475		
SUV_max_	1.012 (1.021–1.189)	0.013*	1.361(1.016–1.824)	0.039*
SUV_mean_	1.017 (0.774–1.336)	0.903		
SUV_peak_	1.009 (0.886–1.149)	0.892		
MTV	0.996 (0.987–1.006)	0.472		
TLG	0.998 (0.993–1.004)	0.569		
SUV_maxwb_	1.000 (0.998–1.002)	0.980		
SUV_meanwb_	1.000 (0.996–1.004)	0.961		
SUV_peakwb_	1.000 (0.997–1.002)	0.981		
MTV_wb_	1.000 (0.999–1.001)	0.943		
TLG_wb_	1.000 (0.999–1.000)	0.601		
LDH, U/L	1.028 (1.012–1.045)	0.001*	1.064(1.020–1.111)	0.004*
Absolute platelet count, × 10^9^/L	1.000 (0.994–1.006)	0.965		
Absolute neutrophil count, × 10^9^/L	1.179 (1.052–1.322)	0.005*	0.936(0.786–1.114)	0.457
Albumin levels, g/L	0.898 (0.802–1.006)	0.062		
dNLR	0.882 (0.656–1.186)	0.407		

*HR* hazard ratio; *95%CI* 95% confidence interval; *IPS* international prognostic score; *EBV* Epstein-Barr virus; *HBV* hepatitis B virus; *SUV*_*max*_ maximum standardized uptake value; *SUV*_*mean*_ mean standardized uptake value; *SUV*_*peak*_ peak standardized uptake value; *MTV* metabolic tumor volume; *TLG* total lesion glycolysis; SUV_*maxwb*_ whole-body maximum standardized uptake value; *SUV*_*meanwb*_ whole-body mean standardized uptake value; *SUV*_*peakwb*_ whole-body peak standardized uptake value; *MTV*_*wb*_ whole-body metabolic tumor volume; *TLG*_wb_ whole-body total lesion glycolysis; *LDH* lactate dehydrogenase; *dNLR* derived neutrophil-to-lymphocyte ratio, defined as absolute neutrophil count/[white blood cell concentration − absolute neutrophil count];*P < 0.05

**Fig. 4:**
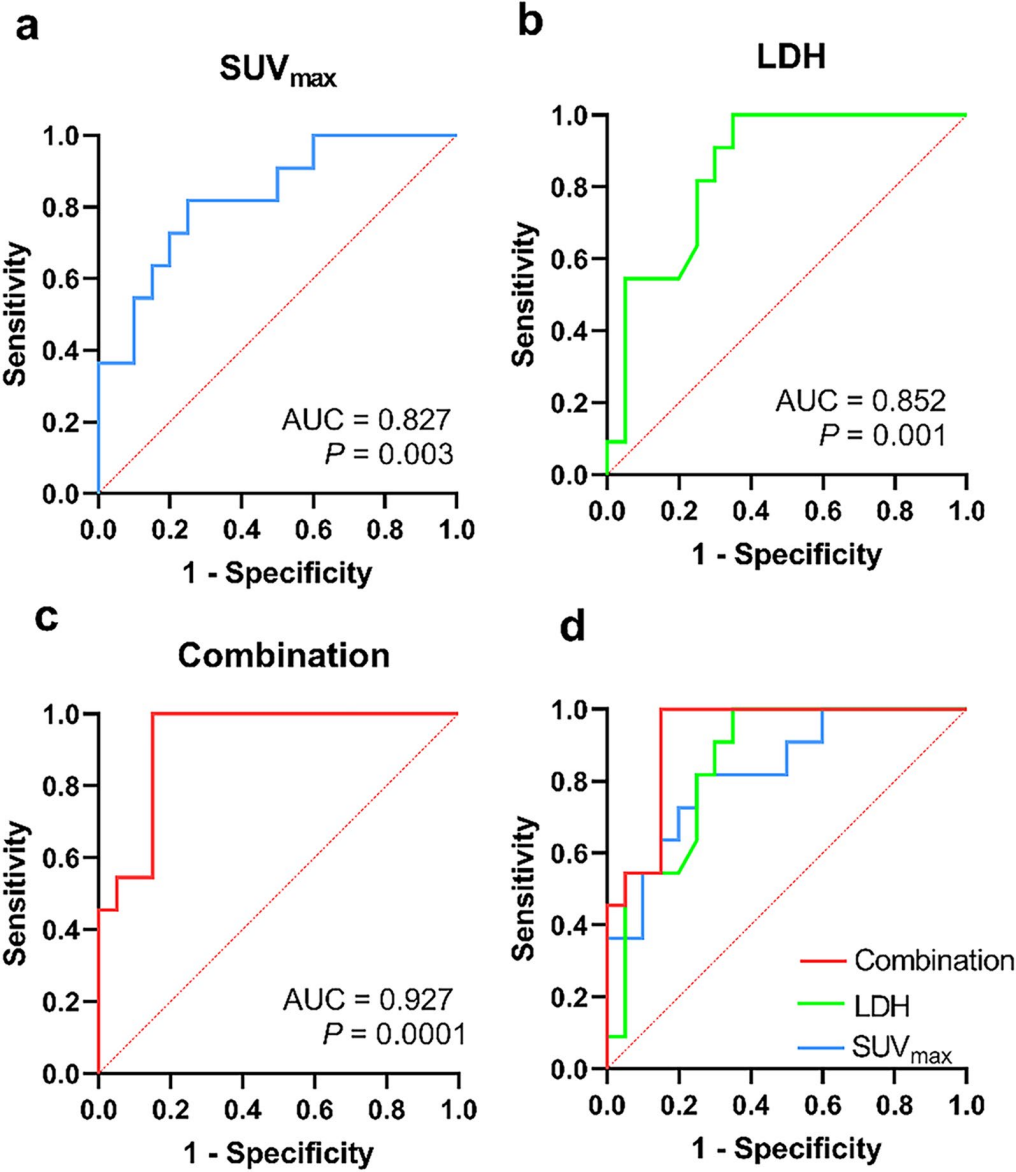
ROC curves (**d**) of SUV_max_ (**a**), LDH (**b**) and their combination (**c**). *AUC* area under curve

We further analyzed the ROC curves for SUV_max_, LDH, and their combination, and found that the AUC value for the combination was greater than that for LDH or SUV_max_ alone (0.927, *P* = 0.0001; 0.852, *P* = 0.001 and 0.827, *P* = 0.003, respectively) (Fig. 4). The Youden index was used to determine the optimal SUV_max_ and LDH cut-off values of 11.62 and 258.5 U/L, respectively. These values were used to categorize the patients into low- (SUV_max_ < 11.62 and LDH < 258.5 U/L), intermediate- (SUV_max_ ≥ 11.62 or LDH ≥ 258.5 U/L), and high- (SUV_max_ ≥ 11.62 and LDH ≥ 258.5 U/L) risk groups.

### Subgroup analysis

**Fig. 5:**
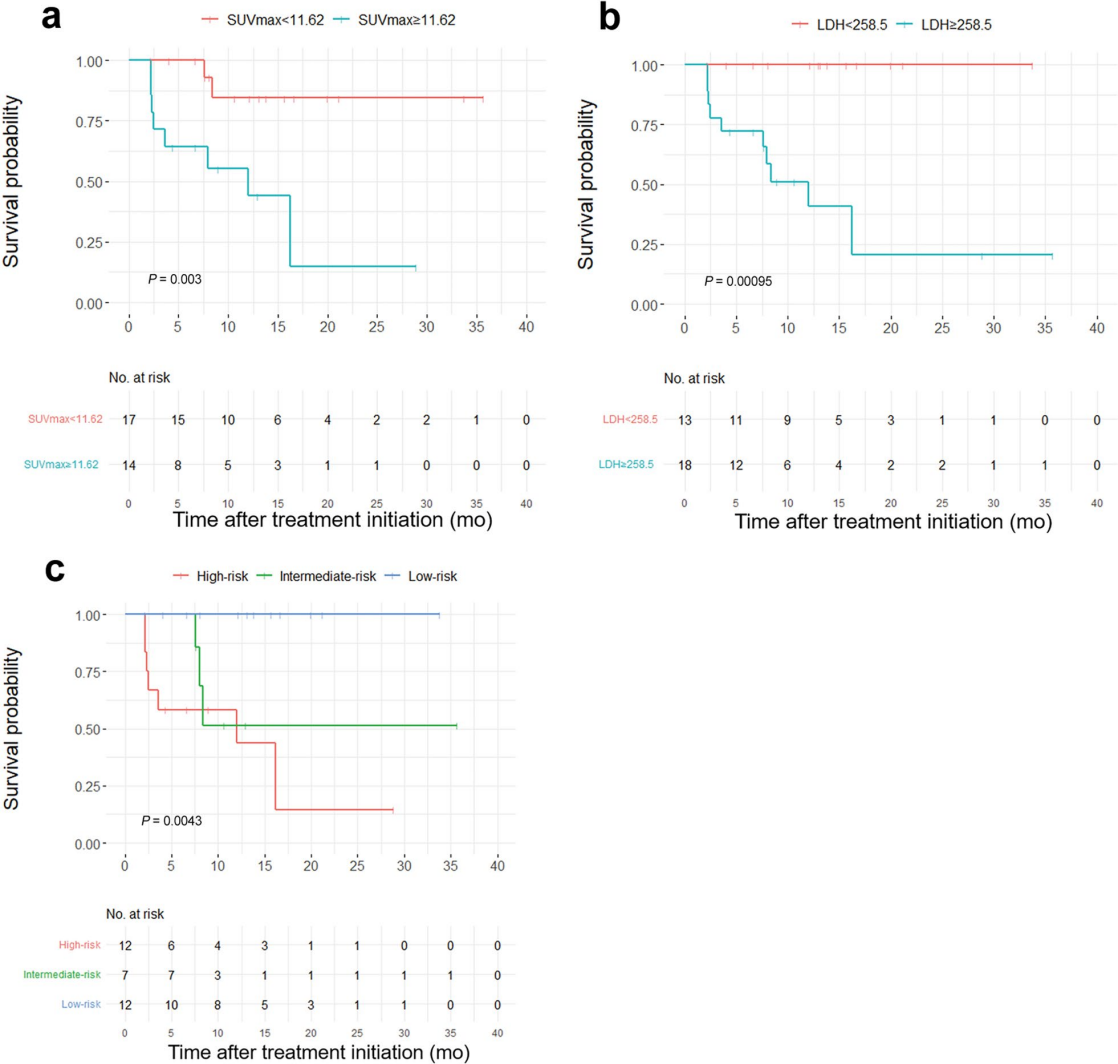
SUV_max_ ≥ 11.62, LDH ≥ 258.5 U/L and high-risk group (SUV_max_ ≥ 11.62 and LDH ≥ 258.5 U/L), were associated with unfavorable outcome. Kaplan–Meier analysis for PFS in the SUV_max_ ≥ 11.62 vs. SUV_max_ < 11.62 (**a**); LDH ≥ 258.5 U/L vs. LDH < 258.5 U/L (**b**); high-risk group (SUV_max_ ≥ 11.62 and LDH ≥ 258.5 U/L) vs. intermediate-risk group (SUV_max_ < 11.62 or LDH < 258.5 U/L) vs. low-risk group (SUV_max_ < 11.62 and LDH < 258.5 U/L) (**c**), respectively

The median PFS was significantly different between patients with SUV_max_ < 11.62 and SUV_max_ ≥ 11.62 (*P* = 0.003). The median PFS was 8 months (95% CI = NA–16.34) in the SUV_max_ ≥ 11.62 group, whereas the SUV_max_ < 11.62 group did not develop disease progression (Fig. 5a). The LDH level (< 258.5 and ≥ 258.5 U/L) was also significantly correlated with PFS (*P* < 0.001). The median PFS was 12 months (95% CI = 7.6–NA) in the LDH ≥ 258.5 U/L group, whereas patients in the LDH < 258.5 U/L group did not show disease progression (Fig. 5b).

There were 12 and 7 patients in the low- and intermediate-risk groups, respectively, and the median PFS was not reached. The remaining 12 patients had a high risk and a median PFS of 12 months. The differences in median PFS between the groups were statistically significant (*P* = 0.0043, Fig. 5c).

## Discussion

ICIs are a promising treatment for R/R cHL. Two anti-PD-1 antibodies, nivolumab and pembrolizumab, have been approved by the United States Food and Drug Administration for use in R/R HL patients who have previously received three or more therapies. However, despite a high ORR, most patients relapse after ICIs monotherapy [7]. Several studies have reported the therapeutic efficacy of anti-PD-1 antibodies combined with chemotherapy [6–8], but there is limited evidence on the factors related to outcomes in these patients. Prognostic factors are essential for personalized therapy and for the identification of patients who may benefit from anti-PD-1 antibody combination therapy. In the present study, we demonstrated that the pretreatment SUV_max_ on ^18^F-FDG PET/CT, LDH levels, and Lugano classification were independent predictors of PFS in R/R cHL patients, indicating the importance of early pretreatment SUV_max_ and LDH assessment.

Previous studies have investigated the HL treatment response and prognosis using ^18^F-FDG PET/CT [9–11], but few studies have investigated the potential role of ^18^F-FDG PET/CT in the response evaluation for combined immunotherapy-chemotherapy. The present study demonstrated that, among the pretreatment ^18^F-FDG metabolic and hematological parameters, SUV_max_ and LDH were the best predictors of the response to combined anti-PD-1 antibody treatment and chemotherapy. Patients with SUV_max_ ≥ 11.62 and LDH ≥ 258.5 U/L had significantly shorter PFS (median: 12 months). The high-risk group included patients with a poor physical condition and high tumor burden. This suggests that immunochemotherapy was less likely to induce a strong immune response for tumor suppression in patients with a high tumor burden prior to the treatment [6, 15]. This may reflect a correlation between inflammation and the metabolic activity of tumors in response to combined ICI and chemotherapy.

Cox multivariable analysis showed that the Lugano classification, SUV_max_, and LDH were independent prognostic factors for PFS. Patients with PD, SUV_max_ ≥ 11.62, and LDH ≥ 258.5 U/L had shorter median PFS (*P* < 0.001) compared to those with CR or SD/PR, SUV_max_ < 11.62, and LDH < 258.5 U/L, respectively. The Lugano classification integrates PET/CT and CT to evaluate the treatment response of lymphomas. LYRIC allows the selection of pseudoprogressive lymphoma patients for immunotherapy, which prolongs survival. A previous study reported that PET/CT assessment based on the Lugano classification or LYRIC predicted the risk of progression and death [16]. In the present study, PD patients were further classified into IR2 and IR3 groups. IR2 patients had worse PFS compared to IR3 patients, but the difference was not statistically significant. This indicated that the occurrence of new lesions may be associated with a worse prognosis.

Tumors with high glycolytic levels (high FDG uptake on PET/CT) consume glucose and produce lactic acid, which leads to PD-1 overexpression in regulatory T cells and the inhibition of CD8^+^ T cells. This contributes to the failure of anti-PD-1 therapy [17]. SUV_max_ is a semiquantitative metabolic biomarker derived from PET/CT and may be used to predict prognosis and treatment outcomes in a noninvasive manner. In the present study, high SUV_max_ values were associated with shorter PFS (*P* = 0.039; hazard ratio = 1.361; 95% CI = 1.016–1.824). However, previous studies of the predictive role of SUV_max_ for PFS and overall survival in HL patients have yielded conflicting results [11, 18–20]. We speculate that the reason for this contradiction is that the patients included in these studies have different therapeutic regimens and most of them are administered with stander-of-care drugs.

Unlike studies that only investigate the prognostic role of metabolic parameters, we combine metabolic parameters with hematological parameters. Previous studies have demonstrated an association between an unfavorable HL prognosis and inflammatory status [12, 13, 21, 22]. LDH is an international prognostic index risk factor related to hypoxia and glycolysis, and it is a strong predictor of survival in invasive lymphoma patients [23–27]. In the present study, the median PFS was significantly different between R/R cHL patients with LDH ≥ 258.5 U/L (median PFS was not reached) and those with LDH < 258.5 U/L (median PFS = 12 months). The combination of ^18^F-FDG PET/CT metabolic and hematological parameters was used to stratify the patients into low-, intermediate-, and high-risk groups; these groups had significantly different PFS values (*P* = 0.0043). High-risk patients may require more intensive surveillance and individualized treatment. Our findings demonstrate the prognostic value of these parameters, which may allow the identification of patients who could benefit from anti-PD-1 combination therapy.

The present study also had some limitations. First, the sample size was relatively small compared to previous studies of standard-of-care drugs. However, a 31-patient population is a moderate one in researches of immunotherapy plus chemotherapy for R/R cHL. Second, PET/CT scans were performed at different institutions, although the quantitative and visual analyses were performed at the same center. In addition, image acquisition and reconstruction were performed in accordance with appropriate guidelines. Third, the course of ICI and chemotherapy, and the type and number of therapies administered before and/or after ICI administration, varied among the patients. Additional studies with a large sample size are required to confirm our findings.

## Conclusions

The present study investigated predictive factors based on imaging and hematological biomarkers. Tumor metabolism, expressed as SUV_max_, reflects intra-tumor necrosis and apoptosis, while LDH indicates tumor inflammation. The combination of metabolic and hematological parameters is a noninvasive and effective predictor of PFS. Additional studies are required to validate our findings for clinical use in R/R cHL patients.

## Data Availability

The data used in the current study are available from the corresponding authors on reasonable request.

## Abbreviations

Anti-PD-1: Anti-programmed-death 1
ORR: Overall response rate
R/R cHL: Relapsed/refractory classical Hodgkin lymphoma
CR: Complete response
PFS: Progression-free survival
ICIs: Immune checkpoint inhibitors
^18^F-FDG PET/CT: ^18^Fluorine-fluorodeoxyglucose positron emission tomography/computed tomography
SUV: Standardized uptake value
MTV: Metabolic tumor volume
TLG: Total lesion glycolysis
EBV: Epstein‒Barr virus
HBV: Hepatitis B virus
LDH: Lactate dehydrogenase
dNLR: Derived neutrophil-to-lymphocyte ratio
LYRIC: Lymphoma Response to Immunomodulatory therapy Criteria
mAbs: Monoclonal antibodies
PR: Partial response
SD: Stable disease
PD: Progressive disease
IR: Indeterminate response
CB: Clinical benefit
NCB: No clinical benefit
IQR: Interquartile range
ROC: Receiver operating characteristic
IPS: International prognostic score
CI: Confidence interval
